# Analysis of the diagnostic efficacy of ultrasound, MRI, and combined examination in benign and malignant breast tumors

**DOI:** 10.3389/fonc.2025.1494862

**Published:** 2025-01-30

**Authors:** Dianpei Ma, Changliang Wang, Jie Li, Xiaohan Hao, Yun Zhu, Zhizhen Gao, Chun Liu, Changfan Luo, Yu Huang

**Affiliations:** ^1^ School of Medical Imaging, Bengbu Medical University, Bengbu, China; ^2^ Department of Radiology, The First Affiliated Hospital of Bengbu Medical College, Bengbu, China; ^3^ College of Information Science and Technology, University of Science and Technology of China, Hefei, China; ^4^ the Research and Development Management Center of Anhui Fuqing Medical Equipment Co., Ltd., Hefei, China; ^5^ Medical Imaging Center, Department of Electronic Science, University of Science and Technology of China, Hefei, China

**Keywords:** magnetic resonance imaging, ultrasound, differential diagnosis, breast cancer, breast tumors

## Abstract

**Background:**

To compare the diagnostic effectiveness of ultrasound (US), magnetic resonance imaging (MRI), and their combined application in distinguishing between benign and malignant breast tumors, with particular emphasis on evaluating diagnostic performance in different breast densities—fatty breast tissue, where fat predominates, and dense breast tissue, which contains a significant amount of fibroglandular tissue.

**Materials and methods:**

A retrospective analysis was conducted on 185 patients with breast tumors, including 90 malignant and 95 benign cases. All patients underwent both US and MRI examinations within one week prior to surgery. The diagnostic accuracy of US, MRI, and their combined use in differentiating benign and malignant tumors was evaluated.

**Results:**

The combined examination demonstrated the highest area under the curve (AUC), sensitivity, and negative predictive value (NPV) (0.904, 90%, 90.4%), outperforming US (0.830, 73.3%, 78.6%) and MRI (0.897, 89.7%, 88.8%). DeLong test results revealed statistically significant differences in AUC between US and MRI, as well as between US and the combined examination (P < 0.05). However, the difference in AUC between MRI and the combined examination was not significant (P = 0.939). In patients with fatty breast tissue, no significant differences were found between MRI and US, or between MRI and the combined examination (P = 0.708 and P = 0.317, respectively). However, the diagnostic performance between US and the combined examination was statistically significant (P < 0.05). For patients with dense breast tissue, the differences in diagnostic performance between US and MRI, and between US and the combined examination, were significant (P < 0.05), while the difference between MRI and the combined examination was not significant (P = 0.317).

**Conclusion:**

MRI and combined examination methods significantly enhance the ability to differentiate benign and malignant breast tumors and provide important clinical value for early breast cancer detection.

## 1 Introduction

Breast Cancer (BC) is the most common cancer among women worldwide and the second leading cause of cancer-related deaths (1). Timely and standardized treatment of benign breast lesions can significantly reduce the risk of cancer. However, BC is challenging to detect in its early stages and is often diagnosed in the middle or late stages, characterized by a high metastasis rate and poor prognosis (1–9). Therefore, accurate identification of breast lesions is of critical clinical importance, as it directly impacts patient management, treatment decisions, and prognosis (2). Recent studies have identified dense breasts as one of the most significant risk factors for breast cancer, with the risk being approximately two times higher in women with dense breasts compared to the average screened population (10, 11). The glandular tissue in dense breasts is more abundant, making it difficult to detect lesions against the background of normal breast parenchyma, as they may be obscured by the normal tissue (12). Consequently, patients with dense breasts require special attention. Mammography is sensitive to microcalcifications, but its sensitivity is significantly reduced in women with dense breasts, ranging from approximately 47.8% to 64.4% (13–16). This can lead to a certain degree of misdiagnosis, potentially causing patients to miss the optimal treatment window, which in turn affects survival rates and quality of life. Additionally, misdiagnosis can contribute to the wastage of medical resources, prolong hospital stays, and increase psychological stress and economic burden. Therefore, reducing misdiagnosis and improving diagnostic accuracy are crucial objectives in clinical practice. Breast magnetic resonance imaging (MRI) has high sensitivity and is not affected by breast density. It can also evaluate extramammary areas, including the contralateral breast, chest wall, axillary region, and lymph nodes (17). It can also detect occult lesions that breast ultrasound (US) and digital mammography cannot identify, aiding in lesion detection and improving the accuracy of early diagnosis, thereby reducing mortality risk. However, it has a high false-positive rate and some contraindications (8, 18, 19). The US has been used at various stages of breast cancer management, including screening dense breasts, diagnosis, chemotherapy, and prognosis, due to its non-invasive nature, lack of ionizing radiation, portability, real time capability to guide biopsies, and cost-effectiveness (20). However, the accuracy of the diagnosis heavily depends on the operator, leading to a large number of unnecessary biopsies and short-term follow-up rates (20–23).

The combined use of US and MRI can fully leverage their complementary advantages, potentially improving the accuracy of differential diagnosis between benign and malignant breast tumors and increasing the early detection rate of malignant tumors. This enables timely intervention, optimizing treatment plans and patient care. Therefore, this study aims to compare the diagnostic efficacy of US, MRI, and their combination in differentiating benign and malignant breast tumors. The focus is on analyzing the diagnostic efficacy of these three methods in different breast densities (fatty and dense breasts). This research hopes to provide more precise and reliable imaging support for BC diagnosis, promoting advancements in early diagnosis and personalized treatment of breast cancer.

## 2 Materials and methods

### 2.1 Study population

**Figure 1:**
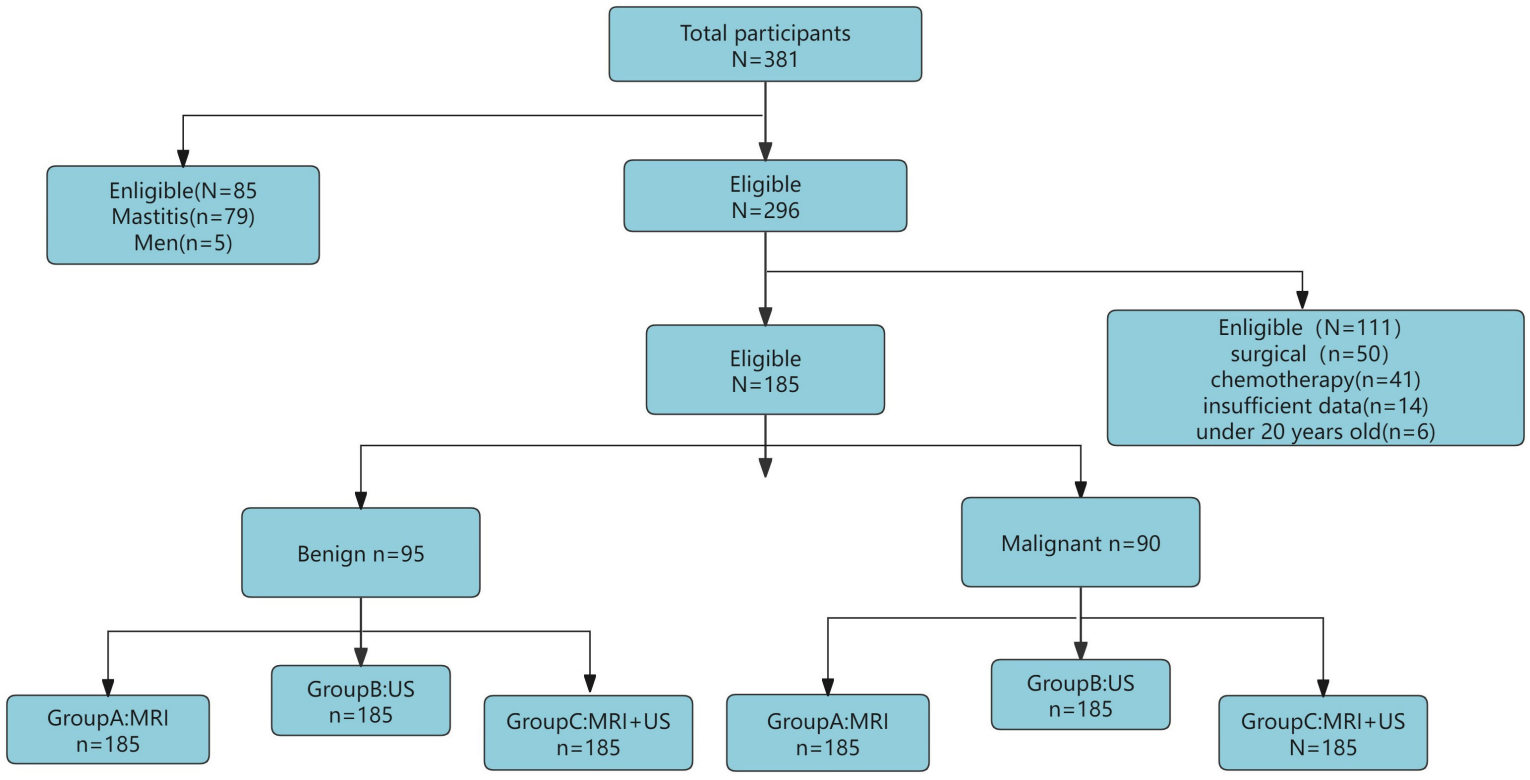
Flowchart showing the patient selection process to form the study sample.

This retrospective study included 381 patients with breast tumors who visited the First Affiliated Hospital of Bengbu Medical University from January 2023 to May 2024. Inclusion criteria: (1) Underwent both US and MRI examinations within one week before surgery; (2) Had not received any breast surgery, radiotherapy, or chemotherapy before the study; (3) Had good quality US and MRI images; (4) Had complete histological characteristics. Exclusion criteria: (1) Age below 20 years; (2) Male patients with breast tumors; (3) Incomplete examination data or missing histopathological features. Ultimately, 185 patients aged 20-75 years (median age 47 years, range 35-54 years) were included in the study. This study adhered to the Declaration of Helsinki and used a retrospective study method, with all patients waiving informed consent. The population selection and trial overview are shown in **Figure 1.**

### 2.2 Study methods

According to the 5th edition of the Breast Imaging Reporting and Data System (BI-RADS) lexicon (24), breast density is classified into four categories: a, b, c, and d. Categories a and b are considered fatty breasts, while categories c and d are considered dense breasts. The diagnostic efficacy of the three methods was considered statistically significant if P was less than or equal to 0.05. Breast density was determined using MRI as the standard, and the nature of the tumor (benign or malignant) was confirmed through pathological examination. All patients underwent both US and MRI examinations within one week before surgery.

### 2.3 Image acquisition

#### 2.3.1 MRI scan sequences

Routine Sequences: T2WI-TRA-Fat Suppression: TR: 3200 ms; TE: 80 ms; Field of View (FOV): 340 x 340 mm; Slice thickness: 4 mm; T1WI-TRA: TR: 5.23 ms; TE: 1.8 ms; FOV: 340 x 340 mm; Slice thickness: 1.2 mm.

Diffusion-weighted Imaging (DWI): Single exponential diffusion-weighted scan (DWI): Diffusion weighting factor (b-values): 0, 1000 s/mm²; TR/TE: 6375 ms/69.8 ms; FOV: 320 mm x 320 mm; Slice thickness: 5 mm.

Dynamic Contrast-Enhanced Imaging: TR: 6.35 ms; TE: 1.3 ms; Slice thickness: 1.2 mm; FOV: 340 x 340 mm; Dynamic enhancement scan with 9 series; Repeat scan 6 times, each scan duration 60 s.

#### 2.3.2 MRI image acquisition

The MRI examinations were performed using a GE Signa 3.0T MRI scanner equipped with a dedicated 8-channel phased-array breast coil. Patients were positioned prone with their feet first, allowing both breasts to naturally hang within the breast coil for imaging. Images were uploaded to the workstation for analysis of lesion characteristics, primarily focusing on size, shape (regular or irregular), and margin clarity. Time-Intensity Curve analysis was conducted to assess lesion enhancement patterns. DWI was processed to generate Apparent Diffusion Coefficient (ADC) maps post-acquisition.

#### 2.3.3 Ultrasound image acquisition

Ultrasound images were acquired in three planes: transverse, coronal, and sagittal, following the BI-RADS lexicon (24) lexicon. The analysis included the evaluation of characteristics such as margin clarity (clear or unclear), shape (regular or irregular), and echogenic pattern.

Both US and MRI images were assessed independently by two breast radiologists with 6 and 10 years of experience in interpreting breast ultrasound images. They were blinded to clinical information and patient age. In cases of disagreement, radiologists discussed the issue and reached a consensus.

According to the BI-RADS lexicon (24) from the American College of Radiology, breast lesions were categorized into 3-5 classes: lesions classified as 3-4a were considered benign, while those classified as 4b, 4c, and 5 were considered suspicious for malignancy.

### 2.4 Pathological analysis

All patients underwent pathological and immunohistochemical examinations. The expression of HER-2 gene immunohistochemistry is categorized as − or + for negative, and +++ for positive. If HER-2 expression is ++, FISH examination is conducted to determine gene amplification, with positive indicating amplification and negative indicating no amplification (25, 26).

### 2.5 Statistical analysis

Statistical analyses were conducted using SPSS Statistics 27.0 (IBM Corp., Chicago, Illinois, USA), R (version 3.5.2), and R Studio (version 1.1.456). Continuous variables with a normal distribution were presented as means with standard deviations, while non-normally distributed variables were represented as medians with interquartile ranges. Categorical data were shown as counts with percentages. The normality of the distributions was evaluated using the Shapiro-Wilk test. For univariate analysis, comparisons of continuous variables were made using Student’s t-test (for normally distributed data) or the Mann-Whitney U test (for non-normally distributed data), while categorical variables were compared using the Chi-square test or Fisher’s Exact test. Sensitivity, specificity, accuracy, and AUC, along with the corresponding 95% confidence intervals (CIs), were computed using the R package report ROC. The DeLong test, a statistical method frequently employed to compare differences between two or more receiver operating characteristic (ROC) curves, was used to evaluate the AUC. This method is particularly valuable for the early diagnosis of breast cancer. A p-value of less than 0.05 was considered statistically significant.

## 3 Results

### 3.1 General information

Out of the 381 enrolled female patients, 79 had mastitis, 6 were male patients, 91 had undergone preoperative surgery and radiotherapy/chemotherapy, 14 had incomplete pathological diagnosis data, and 6 were under 20 years old. Therefore, these were excluded, leaving a final cohort of 185 patients (median age 47 years, range 35-54 years). All patients underwent both US and MRI examinations before surgery, with pathological diagnosis and biopsy results serving as the gold standard.

There are a total of 185 breast lumps among these patients, and their clinical information is shown in **Table 1**. According to BI-RADS classification, BI-RADS 3-4A were assessed as benign lesions, while BI-RADS 4B-5 were assessed as malignant lesions. There were 95 benign lesions, with a median tumor diameter of 1.7 cm (range: 1.2-2.55 cm). These included 69 cases of fibroadenoma, 6 cases of benign phyllodes tumor, and 24 cases of intraductal papilloma. There were 90 cases of breast cancer, with a median tumor diameter of 2.2 cm (range: 1.8-3 cm). Among these, the majority were non-specific invasive carcinoma (74 cases), with 4 cases of invasive lobular carcinoma, 2 cases of ductal carcinoma *in situ*, and 1 case of malignant phyllodes tumor.

**Table 1:** Clinical characteristics of 185 patients.

Characters	Total (n = 185^1^)	Benign (n = 95^1^)	Malignant (n = 90^1^)	P^2^
**Age, Median (IQR)**	47 (35, 54)	37 (27,47.25)	52 (47, 59)	<0.001
**Tumor diameter, Median (IQR)**	2 (1.5, 3)	1.7 (1.2,2.55)	2.2 (1.8, 3)	0.002
History of breast disease n (%)				0.748
Yes	184 (99.5)	95 (100.0)	89 (98.9)	
No	1 (0.54)	0 (0)	1 (1.11)	
Smoking, n (%)				1
Yes	0	0	0 (0)	
No	185 (100)	95 (100)	90 (100)	
History of drinking, n (%)				1
Yes	1 (1)	1 (1)	0 (0)	
No	184 (99)	94 (99)	90 (100)	
Location of the nodule, n (%)				0.501
Left upper outer quadrant	32 (17)	14 (15)	18 (20)	
Left lower outer quadrant	14 (8)	6 (6)	8 (9)	
Left upper inner quadrant	19 (10)	10 (11)	9 (10)	
Left lower inner quadrant	8 (4)	3 (3)	5 (6)	
Central area	17 (9)	11 (12)	6 (7)	
Right Upper outer quadrant	34 (18)	20 (21)	14 (16)	
Right Lower outer quadrant	23 (12)	14 (15)	9 (10)	
Right Upper inner quadrant	27 (15)	10 (11)	17 (19)	
Right Lower inner quadrant	11 (6)	7 (7)	4 (4)	
CEA50, n (%)				0.113
Normal	182 (98)	95 (100)	87 (97)	
Abnormal	3 (2)	0 (0)	3 (3)	
CA199, n (%)				0.486
Normal	184 (99)	95 (100)	89 (99)	
Abnormal	1 (1)	0 (0)	1 (1)	
CA125, n (%)				0.676
Normal	180 (97)	93 (98)	87 (97)	
Abnormal	5 (3)	2 (2)	3 (3)	
CA153, n (%)				0.054
Normal	181 (98)	95 (100)	86 (96)	
Abnormal	4 (2)	0 (0)	4 (4)	
Area, n (%)				0.231
Village	124 (67)	68 (72)	56 (62)	
City	61 (33)	27 (28)	34 (38)	
Menstruation, n (%)				
Under 12 years old	16 (9)	5 (5)	11 (12)	
Over 12 years old	169 (91)	90 (95)	79 (88)	
Menopausal status, n (%)				
Yes	61 (33)	13 (14)	48 (53)	
No	124 (67)	82 (86)	42 (47)	
Parity history, n (%)				
Yes	155 (84)	65 (68)	90 (100)	
No	30 (16)	30 (32)	0 (0)	
Density, n (%)				
Fatty	95 (51.4)	61 (64.2)	34 (46.2)	
Dense	90 (48.6)	34 (37.8)	56 (62.2)	
MRI Morphology, n (%)				
Irregular	89 (48.1)	14 (15.7)	75 (84.3)	
Regular	96 (51.9)	81 (84.4)	15 (15.6)	
MRI border, n (%)				
Unclear	84 (45.4)	12 (12.6)	72 (80)	
Clear	101 (54.6)	83 (87.4)	18 (20.2)	
US Morphology, n (%)				
Unclear	77 (41.6)	37 (38.9)	71 (78.9)	
Clear	108 (58.4%)	58 (61.1)	19 (21.1)	
US border, n (%)				
Irregularity	83 (45.4)	18 (18.9)	65 (72.2)	
Regularity	102 (41.5)	77 (81.1)	25 (27.8)	

^1^Median (IQR) or Frequency (%)

^2^Wilcoxon rank sum test; Fisher’s exact test; Pearson’s Chi-squared test

### 3.2 Diagnostic results of US, MRI, and combined examinations for benign and malignant breast tumors

Among the 185 breast tumors, there were 95 benign tumors and 90 malignant tumors. The diagnostic results are as follows: Benign tumors: US: Detected 88 benign tumors; MRI: Detected 87 benign tumors; US + MRI (Combined): Detected 85 benign tumors; Malignant tumors: US: Detected 66 malignant tumors; MRI: Detected 79 malignant tumors; US + MRI (Combined): Detected 87 malignant tumors.

The diagnostic performance of MRI alone is superior to that of the US alone (P < 0.05). There is no significant difference in diagnostic performance between MRI alone and the combined use of US and MRI (P = 0.052). The combined diagnostic performance of US and MRI is superior to that of US alone (P < 0.05), as shown in **Table 2**.

**Table 2:** Number of cases diagnosed with benign and malignant breast tumors by US, MRI, and combined examinations (n).

Methods	Diagnostic cases (n)	P-Value		
		US	MRI	Combined
**US**		-	0.042	0.038
Malignancy	66 (90)			
Benign	88 (95)			
**MRI**		0.042	–	0.052
Malignancy	79 (90)			
Benign	87 (95)			
**Combined**		0.038	0.052	–
Malignancy	87 (90)			
Benign	85 (95)			

### 3.3 Histological characteristics of 90 cases of malignant breast tumors

**Figure 2:**
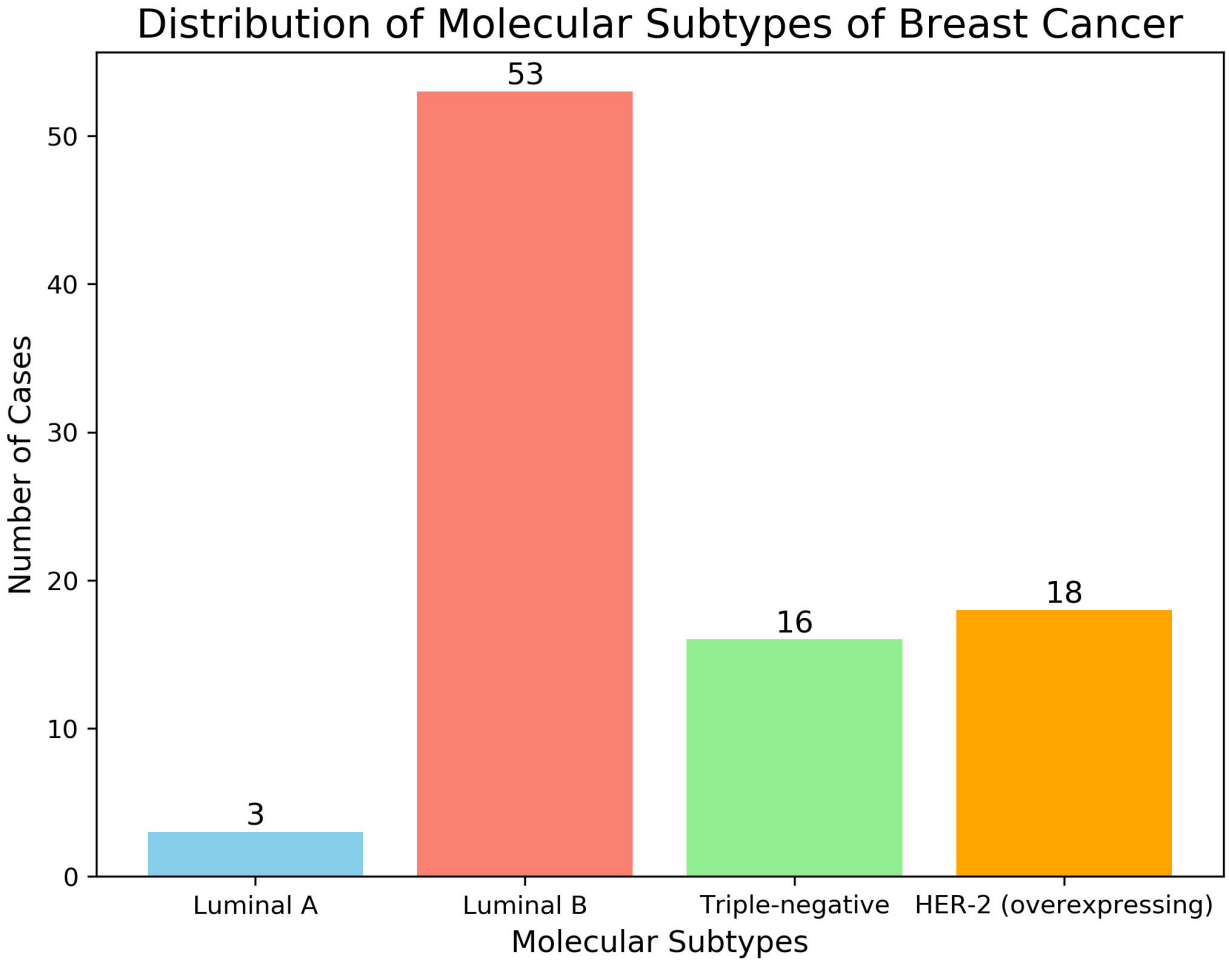
Histological features of 90 cases of breast malignancies.

Pathological and immunohistochemical examinations revealed the following histological subtypes among the 90 malignant breast tumors: Subtype Distribution: Luminal A:3 cases; Luminal B:53 cases; Triple-negative:16 cases; HER-2 overexpression:18 cases; Receptor Status: ER (Estrogen Receptor): ER-: 31 cases (34.4%); ER+: 59 cases (65.6%); PR (Progesterone Receptor): PR-: 32 cases (35.6%); PR+: 58 cases (64.4%); HER-2 (Human Epidermal Growth Factor Receptor 2): HER-2-: 63 cases (70%); HER-2+: 27 cases (30%); These distributions are illustrated in **Figure 2.**

### 3.4 Diagnostic performance of US, MRI, and combined examinations in differentiating benign and malignant breast tumors

The diagnostic performance metrics of US, MRI, and the combined are as follows: US: Accuracy:83.2% [95% CI: 0.773-0.876]; Sensitivity:73.3% [95% CI: 0.634-0.809]; Specificity:92.6% [95% CI: 0.874-0.979]; Positive Predictive Value (PPV):90.4% [95% CI: 0.873-0.972]; Negative Predictive Value (NPV):78.6% [95% CI: 0.71-0.862]; MRI: Accuracy:89.7% [95% CI: 0.857-0.962]; Sensitivity:87.8% [95% CI: 0.81-0.945]; Specificity:91.6% [95% CI: 0.86-0.972]; Positive Predictive Value (PPV):90.8% [95% CI: 0.847-0.969]; Negative Predictive Value (NPV): 88.8% [95%CI:0.825-0.95]; Combined: Accuracy:89.7%[95%CI:0.849-0.935]; Sensitivity:90%[95% CI: 0.838-0.962]; Specificity:89.5% [95% CI: 0.833-0.956]; Positive Predictive Value (PPV):89% [95% CI: 0.826-0.954]; Negative Predictive Value (NPV):89.72% [95% CI: 0.849-0.935]; These results are summarized in **Table 3**.

**Table 3:** Diagnostic efficacy of US, MRI, and combined examinations in differentiating benign and malignant breast tumors.

Variables	US (95%CI)	MRI (95%CI)	Combined (95%CI)
**Sensitivity**	0.733 (0.642-0.825)	0.878 (0.81-0.945)	0.90 (0.838-0.962)
**Specificity**	0.926 (0.874-0.979)	0.897 (0.854-0.936)	0.89 (0.833-0.956)
**PPV**	0.904 (0.873-0.972)	0.908 (0.847-0.969)	0.89 (0.826-0.954)
**NPV**	0.786 (0.71-0.862)	0.888 (0.825-0.95)	0.90 (0.845-0.964)
**Accuracy**	0.832 (0.773-0.76)	0.897 (0.857-0.962)	0.89 (0.857-0.962)

CI, Confidence Interval.

### 3.5 Clinical value of US, MRI, and combined examinations in differentiating benign and malignant breast tumors

The clinical value of US, MRI, and the combined can be assessed using the area under the curve (AUC) values: AUC Values: US:0.830[95% CI: 0.769-0.976]; MRI:0.897 [95% CI: 0.854-0.936]; Combined:0.904 [95% CI: 0.845-0.964]; The differences in AUC between US and MRI, and between US and the combined examination, are statistically significant (P < 0.05).

There is no statistically significant difference in AUC between MRI and the combined examination (P = 0.939, Z = 0.076). MRI alone and combined MRI with the US have superior diagnostic performance compared to the US alone (P < 0.05). There is no significant difference in diagnostic performance between MRI alone and the combined examination (P = 0.052). These findings are summarized in **Table 4.**

**Table 4:** Comparison of diagnostic efficacy of US, MRI, and combined examinations in differentiating benign and malignant breast tumors.

Methods	AUC (95%CI)	P-Value			Z-Value		
		US	MRI	Combined	US	MRI	Combined
**US**	0.830 (0.769-0.976)	**-**	**0.022**	**0.008**	**-**	**2.285**	**2.634**
**MRI**	0.897 (0.854-0.936)	**0.022**	**-**	**0.939**	**2.285**	**-**	**0.076**
**Combined**	0.904 (0.845-0.964)	**0.008**	**0.939**	**-**	**2.634**	**2.285**	**-**

CI, Confidence Interval.

US: Ultrasound. MRI: magnetic resonance imaging. Combined: The combination of the US and MRI.

### 3.6 Diagnostic performance of US, MRI, and combined examinations for different fibroglandular tissue categories in breast density

The diagnostic performance of US, MRI, and their combined use can vary based on different fibroglandular tissue (FGT) categories in breast density. In Fatty Breast Tissue, the diagnostic performance differences between MRI and US alone, and between MRI and the combined examination, are not statistically significant (P = 0.708, P = 0.317 respectively). The difference between the US alone and the combined examination is statistically significant (P < 0.05). In Dense Breast Tissue, the diagnostic performance differences between the US and MRI alone, and between the US and the combined examination, are statistically significant (P < 0.05). There is no statistically significant difference in diagnostic performance between MRI alone and the combined examination (P = 0.317). These results are summarized in **Table 5**.

**Table 5:** Pairwise comparison of FGT classification of benign and malignant breast tumors by US, MRI, and combined examinations at different breast densities.

FGT	Methods	Malignant	Benign	P-Value		
				US	MRI	Combined
**Fatty**	US	33 (17.8%)	62 (33.5%)	_	**0.708**	**0.045**
	MRI	34 (18.4%)	61 (33%)	**0.708**	**_**	**0.317**
	Combined	37 (20%)	58 (31.4%)	**0.045**	**0.317**	**_**
**Dense**	US	40 (21.6%)	50 (27%)	**_**	**<0.001**	**<0.001**
	MRI	53 (28.6%)	37 (20%)	**<0.001**	**_**	**0.317**
	Combined	54 (29.2%)	36 (19.5%)	**<0.001**	**0.317**	**_**

FGT, Fibroglandular Tissue.

US: Ultrasound. MRI: magnetic resonance imaging. Combined: The combination of the US and MRI.

## 4 Discussion

BC originating from the epithelial cells of the mammary gland, is a primary cause of cancer-related deaths in women (27). Research (28) indicates that the five-year survival rate surpasses 95% for patients diagnosed at stages I or II, but significantly drops for those diagnosed at later stages. As a result, accurately distinguishing breast lesions is critically important, as it directly influences patient survival, management strategies, treatment options, and overall prognosis (2). his study therefore examines the diagnostic performance of US, MRI, and their combined use in differentiating benign from malignant breast tumors. It is also the first to investigate the effectiveness of US, MRI, and their combination in distinguishing between benign and malignant lesions.

The results demonstrate that the combined examination of US and MRI achieved the highest values for AUC at 0.904, sensitivity at 90%, NPV at 90.4%, and accuracy at 89.7% (US: AUC 0.830, sensitivity 73.3%, NPV 78.6%, accuracy 83.2%; MRI: AUC 0.897, sensitivity 89.7%, NPV 88.8%, accuracy 89.7%). Both MRI alone and the combined examination showed consistently superior performance compared to the US alone in distinguishing benign from malignant breast tumors. Moreover, the addition of MRI to the US significantly increased sensitivity from 73.3% to 90% in diagnostic assessments. Schmidt et al. (29) found that the PPV of US for residual tumor areas was 77.22%, compared to 74.36% for mammography. Our study demonstrates the PPV of 90.4% for breast US, indicating higher PPV compared to mammography. Jing Chen et al. (30) reported the NPV of 53.98% for breast US, while Schaeffer et al. (31) reported an NPV of 48.1%. In contrast, our study shows an NPV of 78.6% and an accuracy of 83.2%, with the AUC of 0.830 (0.769-0.976), indicating that breast US exhibits good discriminatory performance in distinguishing benign from malignant breast tumors.

These findings provide valuable insights into early detection of breast tumors and personalized treatment strategies. BC poses a serious threat to women’s health globally, and early detection and accurate diagnosis are crucial for improving patient outcomes (9, 30). MRI offers advantages such as high soft tissue contrast and high resolution, potentially providing a new method for distinguishing between benign and malignant breast tumors (32). DWI utilizes the restricted diffusion of water molecules, with malignant tumors typically exhibiting lower ADC values compared to benign tumors (33). Dynamic contrast enhanced (DCE)scanning can obtain semi-quantitative parameters and time-intensity curves, which can better distinguish between benign and malignant breast tumors (34). In future studies, we will place greater emphasis on multi-modal MRI research, specifically analyzing the diagnostic efficacy of DWI and DCE for distinguishing between benign and malignant breast tumors.

The cost-effectiveness of MRI is a widely debated issue. Recent studies have demonstrated that UF-DCE-MRI provides diagnostic performance that is either comparable to or even exceeds that of conventional DCE-MRI (34–43). Kuhl et al. introduced a simplified MRI protocol (44), and in a study involving 443 women and 606 MRI exams, they found that the diagnostic accuracy of the simplified protocol was equal to that of the full protocol. The development of these faster sequences is expected to lower the cost of MRI exams and improve their cost-effectiveness.

In this study, MRI demonstrated an accuracy of 89.7%, sensitivity of 87.8%, specificity of 89.7%, PPV of 90.8%, NPV of 88.8%, and an AUC of 0.897 (0.854-0.936). Compared to US alone, MRI exhibited superior diagnostic performance. When combined with US, MRI showed similar diagnostic results. Our findings suggest that MRI is more effective in distinguishing the nature of breast tumors, underscoring its clinical importance in the early detection of BC.

Early detection is closely linked to improved BC survival rates. Systematic mammography has been used for decades to enhance early BC detection, yet breast cancer continues to be a leading cause of cancer-related deaths among women (45). Research indicates that dense breast tissue is an independent risk factor for BC, potentially masking tumors on mammograms and resulting in missed diagnoses (46–48). Therefore, novel imaging techniques are needed to improve the early differentiation of benign and malignant breast tumors, reduce the likelihood of benign tumors turning malignant, and ultimately lower BC mortality rates.

In this study, we utilized MRI’s high sensitivity, which remains unaffected by breast density, alongside the real-time evaluation offered by US. The combination of both methods yielded promising results. In dense breast tissue, the diagnostic performance of MRI and the combined approach surpassed that of US alone, while the performance of standalone MRI was similar to the combined approach. In fatty breast tissue, the diagnostic results from US and MRI were generally consistent, detecting 66/90 and 79/90 malignant tumors, and 88/95 and 87/95 benign tumors, respectively. In the study by Wienbeck et al. (49), the diagnostic accuracy of contrast-enhanced cone-beam breast CT was assessed in dense breast tissue and compared with non-contrast cone-beam breast CT, mammography, and MRI. The results demonstrated that MRI provided superior accuracy and AUC, with values of 88% and 0.89, respectively. In this study, MRI achieved an accuracy of 89.7% and an AUC of 0.897, aligning closely with the findings of Wienbeck et al. (49). A recent study using a deep learning model based on multimodal imaging for differentiating benign and malignant breast lesions (27) reported an AUC of 0.943 (95% CI 0.792–0.995). In comparison, the AUC for combined US and MRI diagnosis in our study was 0.904 (0.845–0.964), while the AUC for US alone was 0.830 (0.769–0.976). When compared to the performance of combined imaging and deep learning models, the diagnostic effectiveness of US alone was significantly lower. However, the combined diagnostic approach demonstrated accuracy comparable to that of artificial intelligence models, suggesting its potential for providing practical recommendations in the early differential diagnosis of breast tumors.

This study has several limitations. First, it is a single-center study with a relatively small sample size, so future research will involve multi-center, large-scale studies with more extensive sample sizes. Second, the sample distribution is uneven, with malignant tumors predominantly consisting of non-specific invasive breast cancer, which may introduce selection bias. To address this, future studies will include a broader variety of tumor types, such as ductal carcinoma *in situ* and lobular carcinoma. Third, this study included various types of benign and malignant breast tumors, but for the analysis of the diagnostic value of US, MRI, and their combined use, these lesions were simply divided into two groups without further subgroup analysis based on molecular subtypes. Fourth, since this study focused on comparing the diagnostic performance of US, MRI, and combined diagnostics in differentiating between benign and malignant breast tumors, dynamic contrast-enhanced sequences and DWI were not analyzed separately. We plan to explore this aspect in future research. Finally, this study primarily included BI-RADS 3 and BI-RADS 5 lesions, resulting in a larger average tumor size. Future studies will include smaller lesions to validate the findings of this study.

## Data Availability

The original contributions presented in the study are included in the article/supplementary material. Further inquiries can be directed to the corresponding author.
